# How do signals propagate in neuronal compartments? Insights from the Poisson-Nernst Planck model

**DOI:** 10.3389/fncel.2026.1740414

**Published:** 2026-06-05

**Authors:** Paul Paragot, Stella Krell, Claire Guerrier

**Affiliations:** 1 Laboratoire J.A. Dieudonné, CNRS UMR7351, Université Côte d'Azur, Nice, France; 2 CNRS – Université de Montréal CRM - CNRS, Montréal, QC, Canada

**Keywords:** dendritic integration, finite volume method, neuronal plasticity, Poisson-Nernst-Planck model, signal integration

## Abstract

The emergence of novel experimental techniques such as dendritic patch-clamp recordings or genetically-encoded Ca2+-indicators have made the activity of the dendritic tree considerably more tractable, challenging the old postulate that dendrites serve mainly to connect neurons and to convey information with no specific role in synaptic plasticity. Hence, how the dendritic tree transforms synaptic input into neuronal output and defines the relationships between active synapses is now a leading question in neuroscience. To understand the specific role of dendrites, dendritic spines and dendritic tree geometry in shaping neuronal signal, a crucial first step is to understand precisely voltage and ionic dynamics in such small neuronal compartments. For this purpose, we use the Poisson-Nernst-Planck (PNP) model, which is the recognized standard for modeling voltage dynamics and ionic electrodiffusion in electrolytes at the scale now reached by experimental techniques. This non-linear model presents significant challenges for both modeling and simulation due to its high concentration gradients and sensitivity to boundary conditions, making it difficult to simulate on complex geometries. We resolve these issues here by using a state-of-the-art finite volume method, the Discrete-Duality Finite Volume method, which we previously developed to simulate the PNP system of equations on various two-dimensional geometries representing neuronal compartments. Using this method, we investigate the propagation and attenuation of an ionic influx coming from a synapse near a dendritic branch bifurcation and at a dendritic spine, as well as signal invasion in the nearby branches and spines. By connecting these compartments to an ionic reservoir representing the dendritic shaft, we observe that the distance to the shaft strongly influences signal propagation. Notably, a spine positioned close to a large branch behaves as an isolated compartment, while a distant spine is susceptible to signal invasion. Our numerical results therefore suggest that the local geometry of the dendritic tree has a major influence on spine behavior. Consequently, this study proposes that signal integration rules would differ depending on the location of the spine on the dendritic tree. This means that modifications to neuronal structure and organization following activity are not limited to the spine morphology but depend on the entire dendritic tree architecture.

## 1 Introduction

How neuronal dendrites collect, process and transmit information has been a leading question in neuroscience for the past 70 years. Nowadays, the emergence of novel experimental techniques such as dendritic patch-clamp recordings or genetically-encoded Ca2+ -indicators have made the activity of the dendritic tree considerably more tractable. This allowed us to discover its involvement in cellular learning rules and to challenge the old postulate that dendrites serve mainly to connect neurons and to convey information with no specific role in synaptic plasticity (London and Häusser, 2005).

Such technological revolutions in experimental techniques, which are now focusing on reaching the nanoscale, have substantially increased the need for modeling at the same precision. In particular, it is now crucial to carefully develop models describing voltage propagation and ionic dynamics in small neuronal compartments such as small dendritic branches and dendritic spines. Indeed, these compartments include newly formed synapses that are growing or retracting depending on the activity they received and are at the basis of the formation of neuronal networks (Podgorski et al., 2021; Hogg et al., 2022). Inferring the plasticity rules at work, as well as the role of the specific dendritic tree geometry in such synaptic evolution, requires a very fine understanding of the physics underlying ionic and voltage dynamics in these compartments.

Our approach consists in combining state-of-the-art modeling with a robust simulation method to investigate voltage and ionic dynamics in neuronal compartments. The standard model for voltage dynamics in neurons, the Cable theory, dates back to the 1950s, where the theory developed by Lord Kelvin a hundred years before on the propagation of signals on long cables (Kelvin, 1855) was related to signal propagation in nerves. The biophysics underlying this theory was developed over the nineteenth and twentieth centuries to reach its zenith in 1952, with the seminal work of Hodgkin and Huxley on the electrical conduction and excitation in nerves (Hodgkin and Huxley, 1952) and the Nobel Prize in Physiology and Medicine that followed in 1963. The Cable theory makes an analogy between the neuron and an electric wire composed of RC circuits. In this model, the axonal membrane acts as a capacitor in parallel with conductors accounting for ionic channels. The voltage dynamics is represented by a resistive current: the current transported by electrons in the solid structure of the wire follows Ohm's law, where the axial resistance is the axoplasm resistance to the movement of electric charges. This equation is usually coupled to the Hodgkin-Huxley model for voltage-gated ion channels (Hodgkin and Huxley, 1952) to obtain the propagation of an action potential mimicking the action potential observed experimentally (Qian and Sejnowski, 1989; Bower and Beeman, 1998; Carnevale and Hines, 2006). The model writes as a reaction-diffusion equation, i.e., a partial differential equation in space and time. The spatial variable can be replaced by one or several compartments, with the advantage of an easy implementation and fast simulations (Lindsay et al., 2003; Lindsay K. et al., 2007; Lindsay A. et al., 2007), or it can be computed using the continuous framework (Guerrier et al., 2023).

Deriving the Cable equation requires few assumptions, mainly to assume that the voltage is one-dimensional in space and that there are no interactions between the local electric field and the diffusion of ions (Lindsay et al., 2004). Recently, it has been hypothesized that those assumptions don't hold in small microdomains such as filopodia and dendritic spines, in which both the geometry and electrodiffusion play major roles (Cartailler et al., 2017; Savtchenko et al., 2017). Indeed, cytoplasmic conductivity, as defined by the Nernst-Einstein relation, is proportional to a weighted sum of ionic species concentrations. In large axons, variations in concentration due to synaptic activity are small, and hence the conductivity is almost constant. However, in small microdomains, synaptic activity creates significant concentration variations, resulting in substantial changes in cytoplasmic conductivity. At the microdomain level, models taking into account these variations, and more generally electrodiffusion, are usually based on the Nernst-Planck equation describing ionic fluxes, coupled to the Poisson equation describing the voltage, a system of equation referred to as the Poisson-Nernst-Planck (PNP) model (Mori, 2006; Lopreore et al., 2008; Lu et al., 2010; Xylouris et al., 2010; Pods et al., 2013). To summarize, the Cable theory describes voltage propagation at the cellular scale, in one-dimensional homogenized models that consider the cytoplasmic conductivity to be independent of the ionic concentrations. The PNP model is a physics-based approach, describing ionic dynamics and the electric potential at a smaller, sub-cellular scale and in complex geometries (Breit and Queisser, 2021).

The PNP model is derived from the first principle of mechanics, stating mass conservation, and on Maxwell laws for the dynamics of the electric potential. This model, which describes the electrodiffusion of ions in electrolytes, was first described at the end of the nineteenth century by Walther Nernst and Max Planck (Nernst, 1888). It relates the distribution of the ionic concentrations of the different species to the electrical potential through a drift-diffusion flux. Several numerical schemes have been developed to solve the PNP equation in neuronal structures, in one-, two- and three-dimensional geometries, and with various choices of boundary conditions (Eberhardt, 2024; Boahen and Doyon, 2020; Dione et al., 2016, 2019; Boahen and Deteix, 2023). In one-dimensional geometries, various models were developed using or not the hypothesis of electroneutrality (Guerrier et al., 2023; Saetra et al., 2019; Mori, 2006). In two- and three-dimensional geometries, one modeling challenge concerns the tight choice of boundary conditions for the voltage, which can result in the accumulation of ions at the membrane, leading to the formation of a stiff boundary layer with high concentration gradients (Cartailler et al., 2017; Savtchenko et al., 2017). These high concentration gradients perturb the dynamics observed in one-dimensional geometries, and create a numerical challenge, imposing a very high resolution in the PNP simulations to resolve voltage fluctuations close to the membrane. The caveat being that those models are highly demanding in terms of computation, preventing their use at the scale of the dendritic tree.

In this paper, our goal is to actually predict how the influx of ions entering through synapses travels within the dendritic tree and influences nearby neuronal structures. To this aim, we use a framework we previously developed in Paragot et al. (2025), based on the Discrete Duality Finite Volume method, with which we can realize robust and accurate numerical simulations of the PNP model on various complex geometries representing neuronal compartments. We specifically focus on the dynamics of voltage and ionic concentrations within dendrites and dendritic spines in the case of passive membranes. All our results are presented using two generic ions with positive and negative valences that could represent any species, such as sodium, potassium or chloride. Including specific ions and in particular calcium, along with their associated channels, buffers and pump, presents several modeling challenges, and will be the focus of a further study. Here, our goal here is to provide a clear representation of ionic and voltage dynamics in small compartments to improve our understanding of the influence of geometry.

We first consider the propagation of an influx of ions at a dendritic bifurcation. We show that two signals arriving simultaneously at the bifurcation sum linearly (almost linearly in the case of a non-symmetric bifurcation), which is consistent with experimental observations. We then investigate the invasion of a nearby branch by a signal. We test the effects of clustered vs. dispersed synapses on signal integration, and observe a supralinear summation in the case of clustered synapses. We finally determine the distance between the bifurcation and a larger branch that could be the dendritic shaft, such that the latter has a small influence on the dynamics, which is around 20 microns for our geometry. In a second time, we consider dendritic spines and investigate the influence of two neighboring spines on each other when an influx of ions arrives in one spine. It is observed that, contingent on the distance to a larger branch, a dendritic spine can either function as an autonomous compartment or be subject to signal invasion from the neighboring spine. This means that the actual position of the spine on the tree (close to the dendritic shaft or on a small branch at the leading edge of the tree) deeply influences its function.

## 2 Material and methods

### 2.1 The Poisson-Nernst-Planck system of equations

The PNP model describes ionic dynamics and the electric potential at a micro-scale, considering the motion of ions in the cell cytosol due to both diffusion and the electric field generated by the ions, which is called electrodiffusion. The Nernst-Planck equations relate the distribution of the ionic concentrations *c*_*i*_ of species *i* to the electrical potential *V* through the ion conservation equation:


$$\begin{array}{l} \frac{\partial c_{i}}{\partial t}=-\nabla \cdot j_{i} \end{array}$$ (1)

with the drift-diffusion flux *j*_*i*_ being:


$$j_{i}=-\left[D_{i}\nabla c_{i}+D_{i}\frac{z_{i}ec_{i}}{k_{b}T}\nabla V\right].$$

$\frac{D_{i}z_{i}ec_{i}}{k_{b}T}$ is the Einstein relation for the mobility of ion species: *D*_*i*_ is the diffusion coefficient of species *i*, *z*_*i*_ the valence, *k*_*B*_*T* the thermal energy and *e* is the electric charge. This system of equations can then be combined with the Poisson equation:


$$\begin{array}{l} \Delta V=-\frac{1}{\varepsilon }\left(\underset{i}{\sum }z_{i}ec_{i}\right), \end{array}$$ (2)

where ε is the electrical permittivity. The Equations 1 and 2 form the PNP system of equations.

In this paper, we consider two ionic species, *c*_*P*_ and *c*_*N*_, with respective valence *z*_*P*_ = +1 and *z*_*N*_ = −1, on a two-dimensional domain Ω representing a neuronal compartment. These two ionic species represent generic ions, that could be for example sodium, potassium or chloride.

### 2.2 Boundary and initial conditions

For all geometries representing a neuronal compartment, denoted as Ω, the domain boundary ∂Ω is divided into the following types of boundary conditions for the potential and the concentrations:

- At locations where the cell membrane is impermeable to ions, denoted ∂Ω_*r*_, we set a no-flux Neumann boundary condition for *c*_*p*_ and *c*_*N*_: ∀**x** ∈ ∂Ω_*r*_, ∀*t, j*_*P*_(*t*, **x**)·**n** = 0 and *j*_*N*_(*t*, **x**)·**n** = 0, where **n** is the unit outward normal to the domain.
- At a synaptic location ∂Ω_*i*_, we model the influx of ions entering the cell via an AMPA-receptor current *I*_*A*_ (Koch, 1999; Cartailler et al., 2018): if glutamate binds at time *t* = 0, we get
$$\begin{array}{l} I_{A}\left(t\right)=I_{m}\frac{t}{\tau }e^{-\frac{t}{\tau }}. \end{array}$$ (3)
  We consider that only positive ions are entering the cell and thus get at these particular locations a non-homogeneous Neumann boundary condition for *c*_*P*_: $\forall \text{x}\in \partial \Omega_{i},\forall t,j_{P}\left(t,\text{x}\right)\cdot \text{n}=\frac{I_{A}\left(t\right)}{\pi r_{i}^{2}FD_{P}}$, and a homogeneous boundary condition for *c*_*N*_: *j*_*N*_(*t*, **x**)·**n** = 0. Parameters are given in Table 1.
- Everywhere on the cell membrane (on ∂Ω_*r*_ ∪ ∂Ω_*i*_), we impose a no-flux boundary condition for the potential: ∀**x** ∈ ∂Ω_*r*_ ∪ ∂Ω_*i*_, ∀*t*, ∇*V*(*t*, **x**)·**n** = 0.
- To model the junction between our neuronal compartment and the rest of the dendritic tree, we consider that the compartment is connected to a larger branch that acts as an ionic reservoir. Hence, the concentration and potential at these locations, which we denote Γ_*Dir*_, are fixed: $\forall \text{x}\in \Gamma_{Dir},\forall t,c_{P}\left(t,\text{x}\right)=c_{P}^{0}$, $c_{N}\left(t,\text{x}\right)=c_{N}^{0}$ and *V*(*t*, **x**) = *V*^0^.

**Table 1:** Electrodiffusion parameters.

Symbol	Value	Description
*e*	96485 A.s.mol^−1^	Faraday constant
*T*	293,15 K	Temperature
*k*_*b*_	1.38 × 10^−23^ J.K^−1^	Boltzman constant
*F*	96485.33 C.mol^−1^	Faraday constant
ε	7.04 × 10^−10^ F.	Dielectric permittivity
*z*_*P*_	+1	Valence of the cation
*z*_*N*_	-1	Valence of the anion
*D*_*P*_	200 μm^2^.s^−1^	Diffusion coefficient for cation
*D*_*N*_	200 μm^2^.s^−1^	Diffusion coefficient for anion
$c_{P}^{0}$	163 mM	Initial concentration for species *P*
$c_{N}^{0}$	163 mM	Initial concentration for species *N*
*V* ^0^	0 mV	Initial electric potential
*I*_*m*_	300 pA	Maximum of the injected current, Equation 3
τ_*A*_	0.055 s	Decay time constant of the AMPA-receptor current

**Figure 1:**
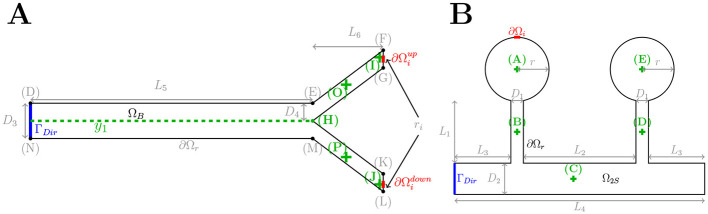
Geometry of the neuronal compartments. **(A)** Bifurcation domain Ω_*B*_, in the configuration *L*_5_ = 11 μm with line *y*_1_ in dashed green, and points of interest in green. The black part of the boundary represents the cell membrane impermeable to ions, the red parts represent the synaptic sites where AMPA-receptors are located, and the blue part represents the connection to a larger branch. **(B)** Domain Ω_2*S*_ representing two neighboring dendritic spines. Black part of the boundary: cell membrane impermeable to ions; red part: synaptic sites where AMPA-receptors are located; blue part: connection to a larger branch.

The dendritic bifurcation domain Ω_*B*_ and the dendritic spine domain Ω_2*S*_ are detailed in Figure 1, including the locations of the various boundary conditions.

Finally, the initial conditions at *t* = 0, defined on **x** ∈ Ω, are:


$$\begin{array}{l} c_{P}\left(t=0,\text{x}\right)=c_{P}^{0},\;c_{N}\left(t=0,\text{x}\right)=c_{N}^{0},\;V\left(t=0,\text{x}\right)=V^{0}, \end{array}$$ (4)

with $c_{P}^{0},c_{N}^{0},$ and *V*^0^ given in Table 1.

### 2.3 Dendritic tree bifurcation

We consider the domain Ω_*B*_ representing the bifurcation, where a large branch divides into two thinner ones (Figure 1A). The detailed geometry is given in Table 2. Note that the main branch has a diameter of 2 μm and the smaller branches have a diameter of 1 μm. This is consistent with the wide extent of scaling laws observed in dendrites (Liao et al., 2021).

**Table 2:** Geometric parameters for domain Ω_*B*_, representing a dendritic bifurcation.

Symbol	Value	Description
*L*_5_	11 μm	Lengths for the sample of the dendrite
*L*_6_	4 μm	Length for both branches
*D*_3_	2 μm	Diameter for the dendrite trunk
*D*_4_	1 μm	Diameter neck for both branches at the junction
*r*_*i*_	0.12 μm	Radius of ∂Ω_*i*_ in Figure 1A for Ω_*B*_
(D)	(0, 2)	Position (x, y) of node D
(E)	(11, 2)	Position (x, y) of node E
(F)	(15, 5)	Position (x, y) of node F
(G)	(15, 4) /(15, 4.2)	Position (x, y) of node G /non-symmetric bifurcation
(H)	(11, 1) /(11, 0.7)	Position (x, y) of node H /non-symmetric bifurcation
(I)	(14.8, 4.6)	Position (x, y) of node I
(J)	(14.8, –2.6)	Position (x, y) of node J
(K)	(15, –2)/(15, –2.6)	Position (x, y) of node K /non-symmetric bifurcation
(L)	(15, –3)	Position (x, y) of node L
(M)	(11, 0)	Position (x, y) of node M
(N)	(0, 0)	Position (x, y) of node N
(O)	(12.9, 3.05)	Position (x, y) of node O
(P)	(12.9, –1.05)	Position (x, y) of node P

The influx of ions is represented by the current *I*(*t*) (Equation 3) injected at the end of the two thin branches ($\partial \Omega_{i}^{\text{up}}$ for the upper branch and $\partial \Omega_{i}^{\text{down}}$ for the lower branch, Figure 1A, red). The connection to the rest of the dendritic tree, represented by a larger dendrite, is at the left end of the large branch (Γ_*Dir*_, Figure 1A, blue). This large dendrite is considered an ionic reservoir due to its large size, i.e., it has fixed ionic concentrations (see Subsection 2.2, Equation 4).

We define five positions along the two small branches, (*I*), (*O*), (*H*), (*P*) and (*J*) to compare the voltage and concentration dynamics in the branches. We investigate the effect of the distance between the bifurcation and the large branch on signal propagation by changing the value of *L*_5_ (Table 3).

**Table 3:** Different coordinates and parameter values that are modified in the three configurations of domain Ω_*B*_: *L*_5_ = 11 μm, *L*_5_ = 22 μm and *L*_5_ = 33 μm.

Description	Config. 1	Config. 2	Config. 3
Value of *L*_5_	11 μm	22 μm	33 μm
Number of triangular cells	6655	7279	7863
*h*	0.36 μm	0.39 μm	0.4 μm
Position (x, y) of node (D)	(0, 2)	(–11, 2)	(–22, 2)
Position (x, y) of node (N)	(0, 0)	(–11, 0)	(–22, 0)

### 2.4 Dendritic spine

We consider the domain Ω_2*S*_ representing two dendritic spines on a dendritic branch (Figure 1B, Table 4).

**Table 4:** Parameters for the domain Ω_2*S*_, representing two neighboring dendritic spines.

Symbol	Value	Description
*r*	0.5 μm	Radius head for both spines
*L*_1_	1 μm m	Length neck for both spines
*L*_2_	1.8 μm	Distance between the two spines
*L*_3_	0.9 μm	Distance between the spine neck and the ionic reservoir.
*L*_4_	4 μm	Total dendrite length
*D*_1_	0.2 μm	Spine neck width.
*D*_2_	0.5 μm	Dendrite width.
*r*_*i*_	0.1 μm	Length of ∂Ω_*i*_
(A)	(0, 1.5)	Coordinates of point (*A*)
(B)	(0, 0)	Coordinates of point (*B*)
(C)	(1, –0.25)	Coordinates of point (*C*)
(D)	(2, 0)	Coordinates of point (*D*)
(E)	(2, 1.5)	Coordinates of point (*E*)

The influx of ions modeling the synapse is located at the top of the left spine (∂Ω_*i*_ in Figure 1B), and the connection to the rest of the tree is at the left end of the dendritic branch (Γ_*Dir*_). The right end represents the end of the branch, with a no-flux boundary condition (∂Ω_*r*_).

We investigate the effect of the distance between the spines and a large branch that acts as an ionic reservoir, representing the connection to the rest of the dendritic tree, by varying parameters *L*_3_ and *L*_4_ (Table 5). Note that the length *L*_4_ represents the total dendritic length, and is therefore the sum of the distances to the reservoir and dendritic termination, as well as the distance between the spines, plus the two spine neck diameters, i.e., *L*_4_ = 2*L*_3_+*L*_2_+2*D*_1_ (Figure 1).

**Table 5:** Parameter values that are modified in the five configurations of domain Ω_2*S*_.

Description	Config. 1	Config. 2	Config. 3	Config. 4	Config. 5
Value for *L*_3_	0.9 μm	2.9 μm	5.9 μm	11.9 μm	23.9 μm
Value for *L*_4_	4 μm	8 μm	14 μm	26 μm	50 μm
Number of triangular cells	2,385	3,365	4,840	7,773	17,548
*h*	0.16 μm	0.16 μm	0.16 μm	0.16 μm	0.16 μm

### 2.5 Simulations

To simulate the PNP system of equations, we use the framework presented in Paragot et al. (2025), where we developed a numerical scheme based on the Discrete-Duality Finite Volume (DDFV) method. The DDFV method allows to work with complex geometries and provides robust, precise numerical results. It is particularly attractive when flows play an important role, as it conserves numerical flows locally. The main idea of the method is to approach the gradient of the solution in all directions, instead of approaching it in only one direction as in classical finite volume methods. In addition to the initial mesh, which is a discrete representation of the geometric domain, the method uses two further discretizations. These are chosen carefully to ensure that the gradient can be fully approximated, with the gradient and divergence operator in discrete duality. This enables the DDFV method to be used on general two-dimensional meshes that do not satisfy the orthogonality condition imposed by classical finite volume methods. This is very practical for doing local refinement, which suits perfectly for problems with stiff boundary layers. The DDFV scheme that we have developed preserves the positivity of the ionic concentrations and enables accurate simulations on complex two-dimensional geometries (Paragot et al., 2025).

We define a time *t*_0_ for each simulation, corresponding to the time at which the concentration *c*_*P*_ is maximal on Ω. Note that it corresponds to a maximum in both space and time. We precise the value of *t*_0_ in each case. We use electrodiffusion parameters as defined in Table Table 1. Finally, we present in this paper the numerical results for *c*_*P*_, as the results for *c*_*N*_ are qualitatively similar. Due to the complex geometries, all meshes are realized using the software *gmsh* and are composed of triangles. The different mesh sizes *h* are given in Tables 3, 4. All simulations are performed with a final time *T*_*f*_ = 1.5 s and a time step *dt* = 5 × 10^−3^ s.

## 3 Results

Using our numerical framework, we examine two types of neuronal compartments: branch bifurcations and dendritic spines. For the dendritic bifurcation, we examine several scenarios: summation of two simultaneous ion influxes, dispersed vs. clustered synapses, propagation of a single ion influx throughout the domain, and the effect of an ionic reservoir on voltage and ion concentration dynamics. Regarding the dendritic spine, we examine how the spine's position relative to a large branch affects its isolation property.

### 3.1 Linear summation of the signal at a bifurcation

We investigate signal summation at a bifurcation in the dendritic tree, i.e., when one main branch divides into two smaller branches. We consider that the connection of the main branch to a larger branch—such as the dendritic shaft—is far (*L*_5_ = 33 μm). We compare the ionic concentrations and the voltage dynamics in two different scenarios: scenario **1** - when only the upper branch receives the ionic influx ($\partial \Omega_{i}^{\text{up}}$ in Figure 1A) and $\partial \Omega_{i}^{\text{down}}$ is turned into a membrane impermeable to ions (homogeneous Neumann boundary condition). Scenario **2**: there is an influx of ions at both ends of the two thin branches ($\partial \Omega_{i}^{\text{up}}$ and $\partial \Omega_{i}^{\text{down}}$ in Figure 1A). We will call a branch 'active' if it receives an influx of ions, and 'inactive' if it does not. We consider the line *y*_1_ at the center of the main branch (Figure 1A, dashed green).

**Figure 2:**
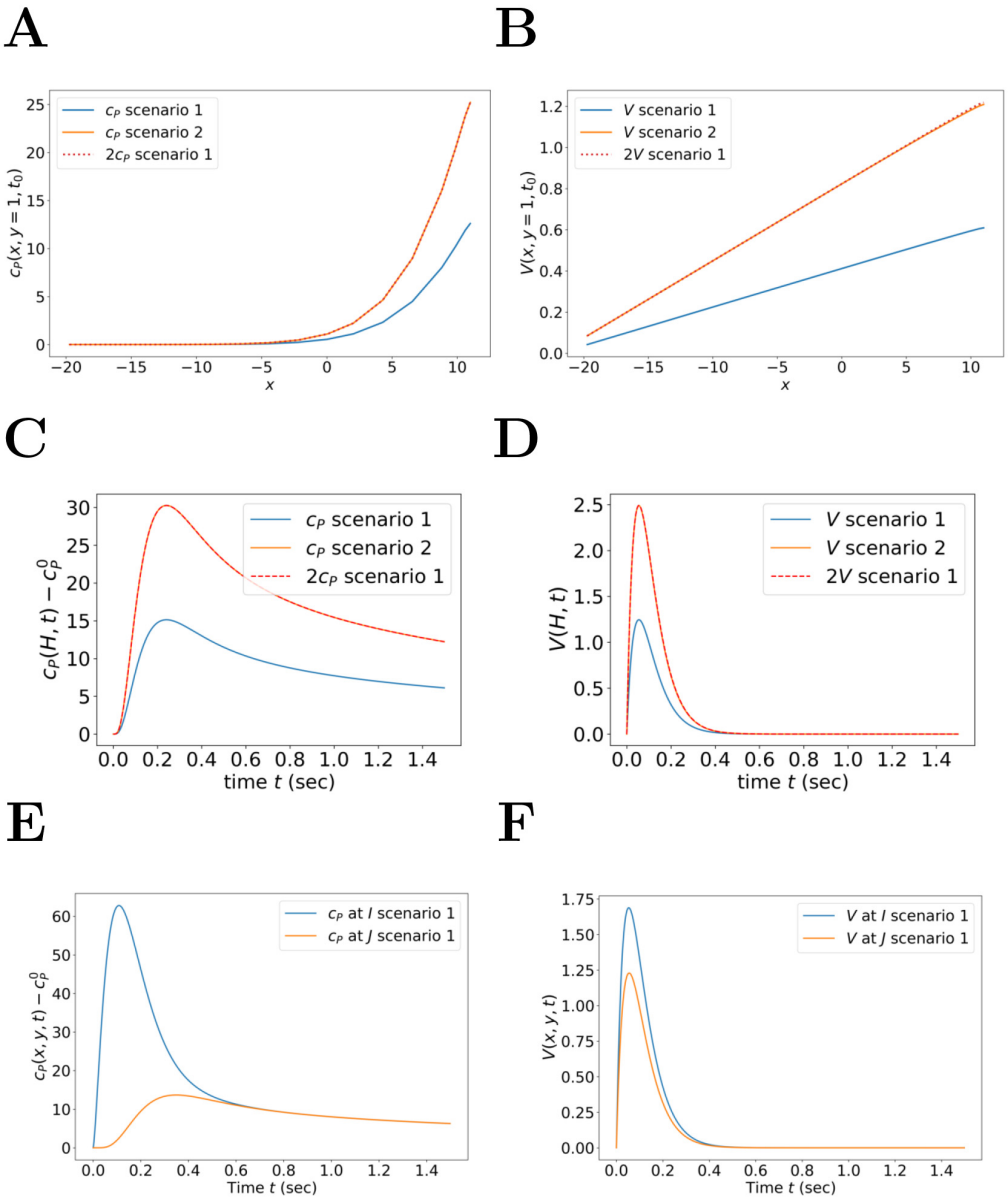
Evolution of the concentration and voltage dynamics on line *y*_1_ for the two scenarios **1** (resp. **2**), with one (resp. two) active branch(es). **(A, B)** Spatial evolution of the concentration $\left(c_{P}-c_{P}^{0}\right)$ **(A)** and potential *V* **(B)**, at peak time *t*_0_ = 0.105 s, for scenario **1** in brown and scenario **2** in blue. In dashed red, we plot twice the values of scenario **1**: $2\left(c_{P}-c_{P}^{0}\right)$ **(A)** and 2*V* **(B)** respectively. **(C, D)** Evolution of the concentration and voltage dynamics at point (*H*) for the two scenarios **1** and **2**. Time evolution of the concentration $\left(c_{P}-c_{P}^{0}\right)$ **(C)** and potential *V* **(D)**, for scenario **1** in brown and scenario **2** in blue. In dashed red, we plot $2\left(c_{P}-c_{P}^{0}\right)$ **(C)** and 2*V* **(D)** at position *H* for scenario **1**. **(E, F)** Time evolution of the dynamics of *c*_*P*_ **(E)** and *V* **(F)** in the small branches for scenario **1** at positions (J) (brown) and (I) (blue).

In Figures 2A, B, we compare the concentration $\left(c_{P}-c_{P}^{0}\right)$ and voltage *V* at time *t*_0_ = 0.105 s for the two scenarios **1** and **2**. In both graphs, the blue curve represents scenario **1** with one active branch, while the brown curve corresponds to scenario **2** where the two branches are active. We observe that, on line *y*_1_, the concentration and potential values in scenario **2** (brown) are twice the values in scenario **1** (blue).

In Figures 2C, D, we plot the time evolution of the concentration $c_{P}-c_{P}^{0}$ and the potential *V* at position (*H*) for the two scenarios with the same colors as before (blue: scenario **1**, brown: scenario **2**). As in Figures 2A, B, we observe that the potential and concentration dynamics in scenario **2** are twice the dynamics in scenario **1**. We computed the absolute difference between the concentration $c_{P}-c_{P}^{0}$ in scenario **2** and twice the concentration $c_{P}-c_{P}^{0}$ in scenario **1** over all times at position *H* and found that the maximal difference is 5.72 × 10^−3^ mM, when the maximum of $c_{P}-c_{P}^{0}$ is 30.26 mM. For the potential, the difference between *V* in scenario **2** and twice *V* in scenario **1**, at position *H*, is 9.95 × 10^−3^ mV, when the maximal value of *V* at position *H* is 2.49 mV.

Similarly, we compute the difference on line *y*_1_ for all times *t*. This difference is zero at the connection for the large branch Γ_*Dir*_ (Dirichlet boundary condition, *x* = - 22 μm, for all *t*), and stays below 10^−2^ mM up to *x* = 8 μm (resp. 10^−1^ mV up to *x* = 8 μm) for the concentration (resp. the potential). It then steeply increases to 0.33 mM (resp. 0.35 mV) for the concentration (resp. the potential) close to *x* = 11 μm [bifurcation point (H)]. In summary, the signal in the large branch is doubled when two branches are active compared to only one active branch. This result is in agreement with the linear summation of inputs in passive membranes observed experimentally.

### 3.2 Branch invasion

We now investigate the invasion of a signal in an inactive branch, by plotting the evolution of the concentration and potential dynamics in the small branches in scenario **1** (only the upper branch is active). We consider the nodes (*I*) and (*J*) toward the end of the upper and lower branch respectively, far from the bifurcation point (Figure 1A and Table 3). In Figures 2E, F, we plot the time evolution of the concentrations $\left(c_{P}-c_{P}^{0}\right)$ (**E**) and the potential *V* (**F**) at the points (*I*) and (*J*) (scenario **1**).

We observe that the influx of ions arriving at the upper branch creates a transient rise in the concentration and in the potential that invades the lower branch. We compare the time and amplitude of *c*_*P*_ and the potential at the two positions (*I*) and (*J*) and observe that the maximum of the signal arrives in (*J*) with a delay of 0.24 s and an amplitude reduced by 78.23 %, decreasing from 62.79 mM in node (*I*) to 13.67 mM in node (*J*) (**E**). Interestingly, the concentration curves overlap at (*I*) and (*J*), for *t*>0.5 s. We hypothesize that this is due to the vanishing of the potential at *t*>0.5 s, highlighting the role of the potential in ionic dynamics (ion transport resulting from the electric field). Concerning the potential dynamics, the maximum of the signal arrives in (*J*) with no delay and a decrease in amplitude of 27 %, decreasing from 1.69 mV to 1.23 mV. In summary, we observe a discrepancy between a rapid and strong invasion of the electrical signal in the inactive branch and a low invasion of the ionic concentration signal, with a delay of a few hundred ms. This result suggests that voltage variations originating from a synapse could travel 'fast and far' throughout the dendritic tree, whereas ionic concentration variations would remain confined to the immediate vicinity of the synapse.

### 3.3 Clustered vs. dispersed synapses

**Figure 3:**
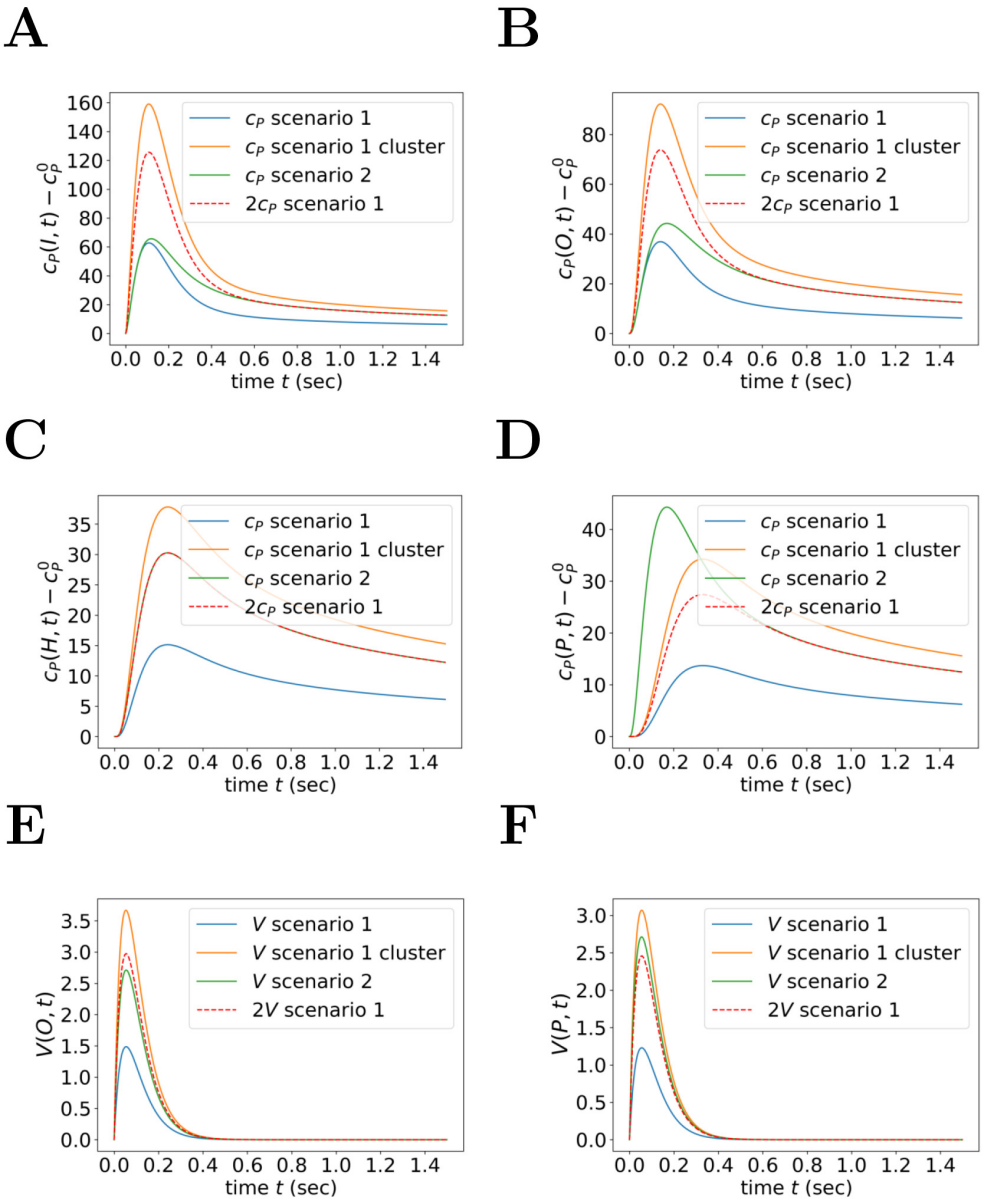
Comparison between clustered vs. dispersed synapses in a branch bifurcation. **(A–D)** Time evolution of the concentration $\left(c_{P}-c_{P}^{0}\right)$ for scenario **1** in blue, scenario **2** (dispersed) in green, twice the values of scenario **1** in dashed red, and the clustered scenario in brown, for the points (*I*), (*O*), (*H*) and (*P*) in the lower and upper branches (Figure 1). **(E, F)** Time evolution of the dynamics of *V* at positions (*O*), and (*P*) for the different scenarios [same as **(A–D)**].

We investigate the effect of clustered synapses vs. dispersed synapses (see Figure 3, (Poirazi et al., 2003a; Kastellakis et al., 2015). We consider a clustered scenario (brown) in which two synapses are located very close ot each other at the end of the upper branch. This is compared with scenario **1** (blue, in which one synapse is activated) and scenario **2** (green, in which one synapse is activated on each branch). Note that scenario **2** corresponds to a dispersed scenario. Finally, we plot in dashed red twice scenario **1**, that represents a linear summation of signals. We plot the time evolution of the concentration in four different points (*I*), (*O*), (*H*), (*P*) located in the upper and lower branches (Table 3 and Figure 1). In the clustered case, we observe that the concentration is 26.66% (resp. 24.98%, 25.01% and 25.36%) higher than twice the concentration in scenario **1** at point (*I*) (resp. (*O*), (*H*) and (*P*)). This corresponds to a supralinear summation of the signal (Figures 3A–D). The same supralinear summation is observed for the voltage (Figures 3E, F).

Note that the linear summation observed in *y*_1_ in the large branch (Figure 2) does not hold inside the upper and lower branches. Indeed, the signals coming from synapses at both ends of the branch only converge at the bifurcation point (*H*).

### 3.4 Quasi-linear summation of the signal at a non-symmetric bifurcation

**Figure 4:**
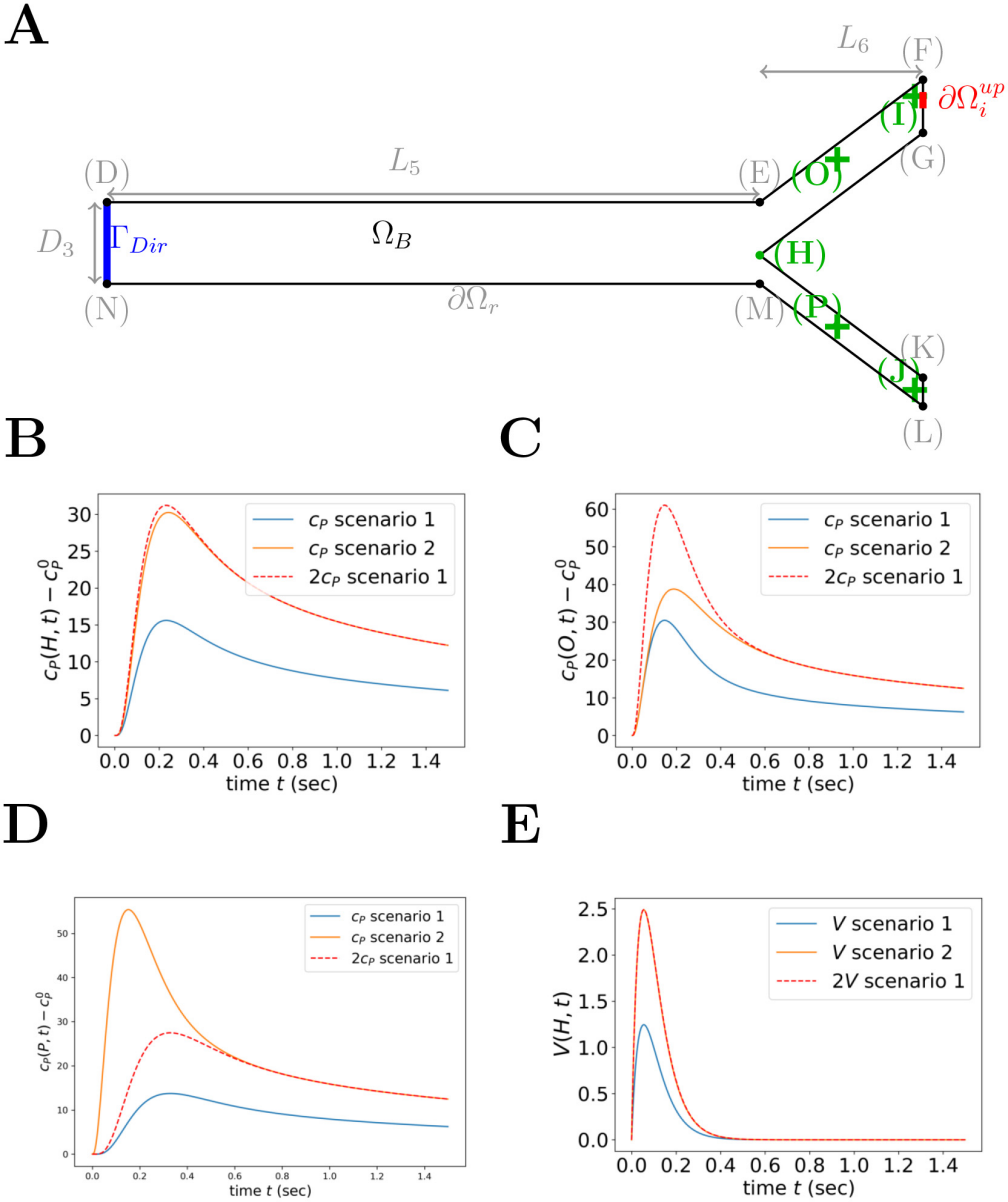
Evolution of the concentration and voltage dynamics for the two scenarios 1 (resp. 2), in the non-symmetric bifurcation domain, with synaptic input in the large branch. **(A)** Schematic representation of the non-symmetric bifurcation. The upper (resp. lower) branch has a width of 0.8 μm (resp. 0.4 μm), the large branch representing the shaft having a width of 2 μm. **(B–E)** Evolution of the concentration dynamics at point (H)- (B), (O)- (C) and (P)- (D) and the voltage dynamics at point (H)–(E), with synaptic input in the upper large branch ∂Ω^*up*^, for scenario 1 (brown), scenario 2 (blue), and twice scenario 1 (dashed red).

We realized simulations similar to those presented in Figure 2, for a bifurcation geometry in which the two small branches have different widths, with a 2:1 ratio (Figure 4). The upper branch has a diameter of 0.8, and the lower branch has a diameter of 0.4. These branches are connected to a large branch with a diameter of 2 (see Figure 4A). To test the linear summation in this case, we performed simulations in three scenarios: when the synaptic input is in the large branch (∂Ω^*up*^) (Figures 4B–E), when the synaptic input is in the small branch (∂Ω^*down*^) data not shown and when there is a synaptic input in both branches. In all cases, we observed that the summation was quasi-linear at the branching point (*H*) (Figure 4B) for the concentration, and perfectly linear for the voltage (Figure 4E). Note that when the synaptic input is in the large branch, the ionic concentration is smaller than when it is located in the small branch. Consequently, the concentration at the junction point *H* is slightly lower than in the scenario where both branches have a synaptic input. The opposite holds true when the input is in the small branch, with a concentration slightly above that in the case of two active branches.

### 3.5 Effect of the distance to an ionic reservoir on voltage and concentration dynamics

Our model considers that the left end of the main branch is connected to a larger one that acts as an ionic reservoir, such that the ionic concentrations and potential at Γ_*Dir*_ are constant. The distance between this ionic reservoir and the bifurcation influences the dynamics of voltage and ionic concentrations everywhere on the domain Ω_*B*_. We expect that this influence is smaller when the length *L*_5_ is increasing, at least close to the bifurcation point. To measure this influence, we apply a current at the end of the two thin branches (∂Ω_*up*_ and ∂Ω_*down*_ Figure 1).

**Figure 5:**
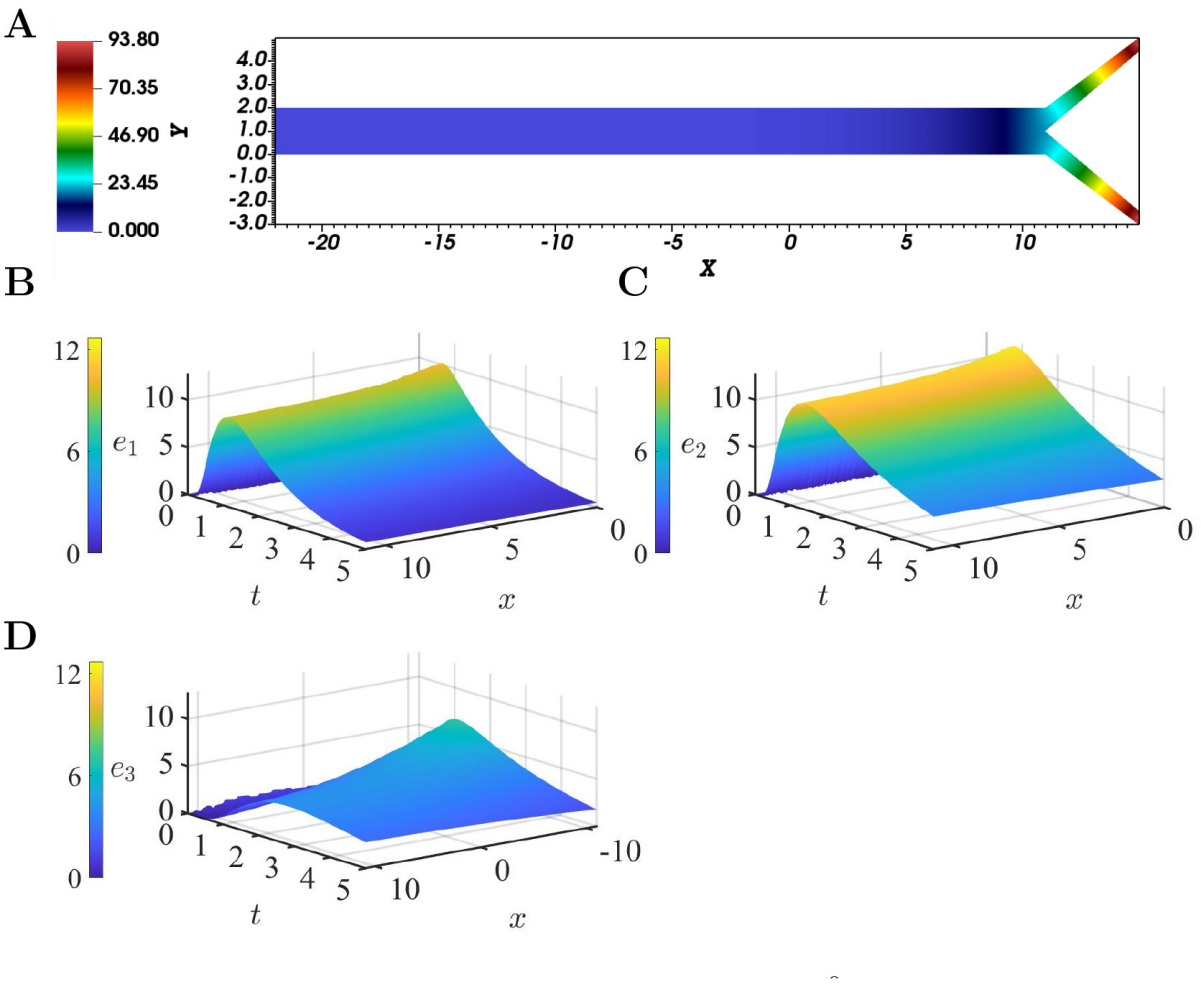
**(A)** Spatial representation of the concentration $c_{P}-c_{P}^{0}$ at time *t*_0_ = 0.105 s, in the case *L*_5_ = 33 μm. **(B–D)** Comparison of the numerical error *e*_1_ (resp. *e*_2_, resp. *e*_3_, Equation 5) between the domains with *L*_5_ = 11 μm and *L*_5_ = 22 μm (resp. with *L*_5_ = 11 μm and *L*_5_ = 33 μm, resp. with *L*_5_ = 22 μm and *L*_5_ = 33 μm) in **(B)** [resp. **(C)**, resp. **(D)**]. We observe the difference between the concentration for the different values of *L*_5_ in time (*t*) and space (*x*).

In Figure 5A, we plot the concentration $c_{P}-c_{P}^{0}$ at time *t*_0_ = 0.105s, in the case *L*_5_ = 33 μm. We observe that the maximum of the solution, 93.8 mM, is reached at the injection boundary ∂Ω_*i*_. Along *x*, the solution decreases to reach the value $c_{P}^{0}$ at the junction with the ionic reservoir (Dirichlet boundary Γ_*Dir*_).

We then realize simulations in domain Ω_*B*_ for three different values of *L*_5_: 11 μm, 22 μm and 33 μm. Note that for each value of *L*_5_, the domain is modified, and so is the mesh. We indicate the different coordinates and parameter values that are modified in the three configurations of domain Ω_*B*_ in Table 3. We compare the numerical solutions obtained on the different domains by evaluating their difference on the straight lines *y*_1_ (Figure 1A). We thus compute the difference between each numerical solution and consider this difference relative to the peak amplitude. We note (*e*_*p, q*_) the absolute value of the difference for *c*_*P*_, on line *y*_1_, for the domains with *L*_5_ = *p* and *L*_5_ = *q*:


$$\begin{array}{l} e_{p,q}=|c_{P,p}-c_{P,q}|. \end{array}$$ (5)

We plot the results in Figure 5: *e*_1_: = *e*_11, 22_ (**B**), *e*_2_: = *e*_11, 33_ (**C**) and *e*_3_: = *e*_22, 33_ (**D**). We consider that the influence of the Dirichlet boundary condition on ionic and voltage dynamics is small when this relative difference is below 3.6%.

We observe that the difference between the solutions is always maximal at the junction with the larger branch (Dirichlet boundary Γ_*Dir*_) at *x* = 0 for *e*_1_ and *e*_2_ or at *x* = −11 for *e*_3_. The maximal difference between the concentration values *c*_*P*_ for *L*_5_ = 11 μm and *L*_5_ = 22 μm is 10.05 mM (**B**). For *L*_5_ = 11 μm and *L*_5_ = 33 μm, the maximum difference is 12.87 mM (**C**), and finally, for *L*_5_ = 22 μm and *L*_5_ = 33 μm, it is 6.36 mM (**D**). The surfaces in **B, C** look similar, with a plateau-like shape along *x* of the maximum, whereas the curve in **D** shows a decrease, with smaller values. Finally, we conclude that, as expected, the influence of the ionic reservoir (Γ_*Dir*_) on the concentration dynamics *c*_*P*_ decreases when the length *L*_5_ of the main branch increases. Specifically, for our geometry, the influence is small if the reservoir is at a distance larger than 22 μm. This result demonstrates the limit behavior of branch invasion when the ionic reservoir is “far.” The presence of a reservoir acts as a sink. In our geometry, the effects of this sink disappear if the branch exceeds twenty micrometers in length. Otherwise, the sink reduces the amplitude of the voltage and concentration signals.

### 3.6 Influence of the distance between an ionic reservoir and a dendritic spine on the spine isolation properties

We consider two neighboring dendritic spines and investigate the influence of a signal arriving in one spine on the ionic and voltage dynamics of the other. We test the hypothesis that dendritic spines act as autonomous compartments, isolating the material located at their head from the rest of the dendritic tree (Hering and Sheng, 2001), vs. the hypothesis of signal invasion, when the voltage and concentration in a spine are substantially increased by an ionic influx arriving in a neighboring one.

We consider the domain Ω_2*S*_ representing two neighboring dendritic spines (Figure 1B), with parameters given in Table 4. Each spine has a head represented by circles with centers (*A*) and (*E*), and a neck represented by a thin vertical rectangle. They are both connected to a dendrite, which is a large horizontal rectangle with points (*B*), (*C*) and (*D*). Within one of the spines, which we call the active spine, we model an influx of ions at boundary ∂Ω_*i*_ (non-homogeneous Neumann boundary condition, see Subsection 2.2 and Equation 3). The spine that does not receive an influx of ions is called the inactive spine. We address the questions of the distance at which the influx of ions propagates from the active spine and to what extent the inactive spine perceives this influx. In particular, we investigate the effect of the proximity of a larger branch, such as the dendritic shaft, on concentration and potential dynamics, i.e., we investigate the effect of the distance *L*_3_ between a spine and the ionic reservoir (Dirichlet boundary Γ_*Dir*_) on voltage and ionic dynamics. We consider several domains with *L*_3_ ranging from 0.9 μm to 23.9 μm. In each configuration, the length *L*_4_ is modified such that *L*_4_ = 2*L*_3_+2*D*_1_+*L*_2_, and *L*_2_ is set to 1.8 μm (Figure 1B). The different mesh information is given in Table 5.

**Figure 6:**
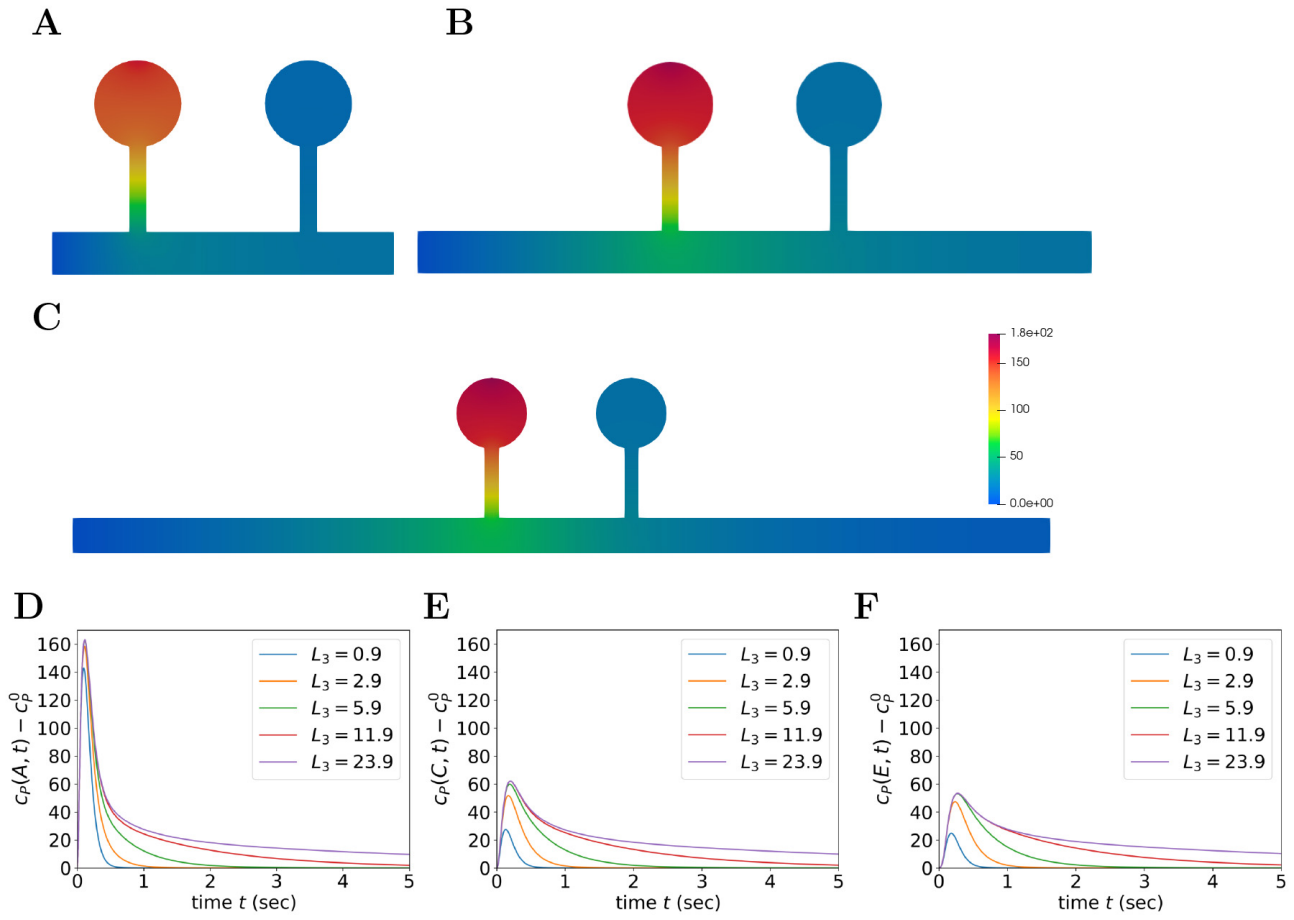
**(A–C)** Spatial representation of the concentration *c*_*P*_ in domain Ω_2*S*_, with *L*_3_ = 0.9 μm **(A)**, *L*_3_ = 2.9 μm **(B)** and *L*_3_ = 5.9 μm **(C)** at *t*_0_ = 0.1 s. The range of the colorbar is fixed between plots, with a maximum value $c_{P}-c_{P}^{0}=180.2$ mM, corresponding to the maximum of $c_{P}-c_{P}^{0}$ in the configuration with *L*_3_ = 5.9 μm. **(D–F)** Time evolution of the concentration $\left(c_{P}-c_{P}^{0}\right)$, for different values of *L*_3_ ranging from 0.9 to 23.9 μm, at positions (A) **(D)**–(C) **(E)**–(E) **(F)**.

Figures 6A–C illustrates the influence of the length *L*_3_ on the dynamics of $c_{P}-c_{P}^{0}$. We plot $c_{P}-c_{P}^{0}$ at times *t*_0_ = 0.1 s for *L*_3_ = 0.9 μm, 2.9 μm and 5.9 μm. Note that the minimum value for $c_{P}-c_{P}^{0}$ is imposed by the Dirichlet boundary condition at Γ_*Dir*_ ($c_{P}-c_{P}^{0}$ = 0 mM, with $c_{P}^{0}$ = 163 mM, Table 1). The range of the colorbar is fixed between plots, with a maximum value $c_{P}-c_{P}^{0}=180.2$ mM, corresponding to the maximum of $c_{P}-c_{P}^{0}$ in the configuration with *L*_3_ = 5.9 μm.

We observe that the size of the domain influences the values of the concentration within the two heads, with a larger *L*_3_ leading to a higher concentration in the active spine and a lower concentration value in the inactive one. We also observe a slight increase in $c_{P}-c_{P}^{0}$ peak time when *L*_3_ increases. Hence, the results suggest that the farther the spine is from a large ionic reservoir, the farther the signal propagates.

We then investigate the propagation of the concentration within the dendrite and inactive spine at several specific points (A)–(E) (Figure 1, Table 4) in the five different configurations (*L*_3_ ranging from 0.9 μm to 23.9 μm). We plot in Figures 6D–F the time evolution of the concentrations $\left(c_{P}-c_{P}^{0}\right)$. We observe the effect of the ionic reservoir on the time evolution of the concentration. As expected, the shorter *L*_3_, the bigger the impact on signal propagation. Indeed, for *L*_3_ = 0.9 μm (red), we observe that the max of $c_{P}-c_{P}^{0}$ goes from 142.80 mM in (A) to 81.22 mM (resp. 27.53 mM, 25.13 mM, 24.77 mM) in (B) [resp. (C), (D) and (E)]. We define the ratio of signal reaching (*E*) as $r_{A-E}^{c}=\frac{\underset{t\in \left[0,T_{f}\right]}{\max}\left(c_{P}\left(E,t\right)-c_{P}^{0}\right)}{\underset{t\in \left[0,T_{f}\right]}{\max}\left(c_{P}\left(A,t\right)-c_{P}^{0}\right)}$. In this case, this ratio is 17%, meaning that the signal does not propagate inside the dendrite and the inactive spine. Hence, for short *L*_3_, the active spine behaves as an autonomous compartment, isolating its material from the rest of the dendritic tree.

While increasing *L*_3_ to 2.9 μm, the max of $c_{P}-c_{P}^{0}$ goes from 158.41 mM in (A) to 102.31 mM (resp. 51.66 mM, 47.65mM and 47.31 mM) in (B) [resp. (C), (D) and (E)]. Hence, the ratio of signal reaching (*E*) is 30 %. Increasing again *L*_3_ to 5.9 μm and 23.9 μm, a threshold seems to emerge, with 33 % of the peak concentration in (A) transmitted to (E). This means that if the ionic reservoir is far enough, then up to 30% of the concentration signal will influence a neighboring spine. Note that we also observe a shift in the peak time between position (*A*) and (*E*), denoted by $\Delta_{A-E}^{c}$, going from 0.09 s for *L*_3_ = 0.9 μm to 0.13 s for *L*_3_ = 2.9 μm and 0.17 s for *L*_3_ = 23.9 μm. The peak time of the concentration signal at position (*A*) for *L*_3_ = 23.9 μm is *t*_0_ = 0.110 s.

Finally, concerning the voltage, we observe that the ratio of the signal reaching (*E*), $r_{A-E}^{V}=\frac{\underset{t\in \left[0,T_{f}\right]}{\max}\left(V\left(E,t\right)\right)}{\underset{t\in \left[0,T_{f}\right]}{\max}\left(V\left(A,t\right)\right)}$, goes from 28% to 71%, and even 91% for *L*_3_ = 0.9 μm, resp. *L*_3_ =5.9 μm and resp. *L*_3_ =23.9 μm. The shift in the peak time between the electrical signal in (*A*) and the electrical signal in (*E*), denoted by $\Delta_{A-E}^{V}$, is below 0.005 s in all curves, when the peak time of the voltage signal at position (*A*) for *L*_3_ =23.9 μm is *t*_0_ = 0.05 s. This shows that the voltage signal propagates faster and with a higher amplitude compared to the concentration one.

Table 6 gives, for each configuration, the time shift $\Delta_{A-E}^{c}$, the peak amplitude $\underset{t\in \left[0,T_{f}\right]}{\max}\left(c_{P}\left(E,t\right)-c_{P}^{0}\right)$ at position (*E*) and the ratio $r_{A-E}^{c}$ of signal reaching (*E*) for the concentration dynamics, as well as the time shift $\Delta_{A-E}^{V}$, the peak amplitude $\underset{t\in \left[0,T_{f}\right]}{\max}\left(V\left(E,t\right)\right)$ at position (*E*) and the ratio $r_{A-E}^{V}$ of signal reaching (*E*) for the potential dynamics.

**Table 6:** Time shift $\Delta_{A-E}^{c}$ in the concentration signal, peak amplitude $\underset{t\in \left[0,T_{f}\right]}{\max}\left(c_{P}\left(E,t\right)-c_{P}^{0}\right)$ of the concentration at position (*E*), ratio $r_{A-E}^{c}$ of the concentration signal in (*A*) reaching the inactive spine (*E*), time shift $\Delta_{A-E}^{V}$ of the electrical signal, peak amplitude $\underset{t\in \left[0,T_{f}\right]}{\max}\left(V\left(E,t\right)\right)$ at position (*E*) and ratio $r_{A-E}^{V}$ of the electrical signal from (*A*) reaching (*E*), for the five different configurations with *L*_3_ going from 0.9 μm to 23.9 μm.

*L*_3_	$\Delta_{A-E}^{c}$	$\underset{t\in \left[0,T_{f}\right]}{\max}\left(c_{P}\left(E,t\right)-c_{P}^{0}\right)$	$r_{A-E}^{c}$	$\Delta_{A-E}^{V}$	$\underset{t\in \left[0,T_{f}\right]}{\max}\left(V\left(E,t\right)\right)$	$r_{A-E}^{V}$
0.9 μm	0.09 s	24.77 mM	17 %	0.005 s	0.25 mV	28 %
2.9 μm	0.13 s	47.31 mM	30 %	0.005 s	0.75 mV	55 %
5.9 μm	0.16 s	53.09 mM	33 %	0.005 s	1.52 mV	71 %
11.9 μm	0.17 s	53.35 mM	33 %	0 s	3.11 mV	84 %
23.9 μm	0.17 s	53.35 mM	33 %	0.005 s	6.32 mV	91 %

This indicates that in this condition, a signal arriving in a spine is influencing the ionic concentration and voltage in its inactive neighbors, which we call a signal invasion. The voltage invasion is more important than the ionic concentration invasion. To summarize, our simulations suggest that depending on the distance to the closest ionic reservoir, a spine can either act as an autonomous compartment isolated from its neighbors or be subject to signal invasion.

## 4 Discussion

In this paper, we investigate voltage and ionic concentration dynamics in small neuronal compartments with passive membranes. In particular, we are interested in signal propagation and its influence on nearby neuronal structures. To this aim, we realize numerical simulations of the Poisson-Nernst Planck system of equations that models electrodiffusion at the sub-cellular scale. We consider two-dimensional geometries representing neuronal compartments and use the DDFV method, an advanced Finite Volume method that allows us to realize accurate simulations of this highly non-linear system in any type of geometry. This method has the advantage of locally conserving the numerical fluxes, which is fundamental when simulating physics-based models. It also approaches these fluxes in all directions, leading to a more accurate representation of ionic dynamics, especially in zones of high gradients. Finally, the method breaks free from the traditional constraints on the discretization of the domain (that require the use of admissible conformal meshes), allowing the representation of complex geometries.

The PNP system is a non-linear system of partial differential equations, which makes analytical computations of signal attenuation/summation in two- or three-dimensional complex domains extremely difficult. To do some analysis, most studies represent the dendritic branches as cylinders, and use a symmetry argument to reduce the three-dimensional system to a one-dimensional one. Equivalent electrical models for a single branch or for a spine can in this case be derived, with or without considering electrodiffusion (Cartailler and Holcman, 2018; Lindsay et al., 2004). Analysis at the scale of the dendritic tree can be done using the “sum-over-path” approach, where the fundamental solution of the Poisson equation is derived in a one-dimensional graph (Bressloff et al., 1996). Nevertheless, this approach does not include the non-linear coupling to the Nernst-Planck equations, and the development of the method have only been done so far for linear operators. Hence, to answer the question of signal propagation in complex neuronal geometries, we chose to use a simulation approach.

In our previous paper (Paragot et al., 2025), we proposed a non-linear scheme for the PNP system of equations using the DDFV method, with the additional property that it preserves the positivity of ionic concentration. We assessed the accuracy of our DDFV scheme through a series of test cases and obtained numerically a second-order accuracy in space, which is comparable to the convergence order of the Poisson and Nernst-Planck equations alone. We then showed that the bulbous head of the dendritic spine requires simulations of a two-dimensional geometry to investigate its specific voltage and ionic dynamics, meaning that the traditional Cable theory is not well-suited to study dendritic spines at the sub-cellular level. This is in accordance with other numerical studies comparing the PNP and Cable models in various geometries (Breit and Queisser, 2021). We finally investigated the effect of the distance between two spines on signal invasion. We showed that dendritic spines can sense electrical signals farther away than concentration signals, making a clear distinction between these two types of signals following synaptic activity. A direct consequence of this is that calcium imaging is not a good marker of electrical activity in these small neuronal structures, emphasizing the need to develop voltage sensors. We use the same strategy and numerical scheme in the present paper.

In this study, we first investigate signal summation at a dendritic branch and show that, in the absence of any ionic channels other than synaptic ones, two signals arriving simultaneously sum almost linearly. This result is consistent with a linear summation of the signal in passive dendrites observed experimentally (Cash and Yuste, 1999), and is due to the passive properties of the membrane. It should be noted that, in the case of two dendritic branches of the same diameter connected to a large branch, the summation is perfectly linear. However, for dendritic branches of different diameters, the summation is almost linear. Note that we are considering passive membranes, and that the addition of voltage-gated ionic channels and pumps would significantly change the result. We also investigate signal invasion from one branch receiving a synaptic signal to a nearby passive branch. We observe that the electrical signal rapidly and strongly propagates, with 69% of the signal reaching the other branch, whereas the concentration signal has an amplitude reduced by 82%, leaving only 18% of the signal on the receiving branch, with a significant delay of hundreds of ms (210 ms for our specific simulation). In terms of dynamics, this means that in the dendritic tree, when one branch receives a signal, the nearby branches will sense the voltage shift and its consequences, such as those affecting low-threshold voltage-gated channels. However, they will not sense ionic concentration changes very well. A future study including all ions and dendritic mechanisms will be needed to assess this prediction, but as a first guess, our study predicts that voltage-induced changes would be triggered in nearby branches, whereas calcium-induced changes would not.

This result is similar to our previous result focusing on dendritic spines, which highlights that small dendritic branches act as neuronal compartments and that in small passive parts of the dendritic tree, voltage sensors are required to understand the precise consequences of voltage variations on nearby neuronal structures. We then test the effect of synapse distribution on signal propagation by comparing clustered and dispersed synaptic signals. We observe that a cluster of synapses exhibits supralinear summation for both concentration and voltage. This is consistent with experimental findings indicating that the activation of nearby synapses exhibits supralinear summation, whereas the activation of synapses in different branches exhibits linear summation (Poirazi et al., 2003b; Polsky et al., 2004; Kastellakis et al., 2015). This demonstrates that non-linear dendritic integration, which is essential for various neural computations such as feature detection, can occur in passive membranes. This is a first step toward understanding the complex rules of the neural computations of single neurons (Spruston and Kath, 2004).

Our final result concerns the influence of an ionic reservoir on voltage and concentration dynamics. This reservoir can represent any large branch to which the neuronal compartment is connected to. The rationale behind this is that because the large branch is so large compared to the thin branch, we can consider the ionic concentrations and voltage at the junction to be constant. In the equations, the reservoir is modeled using a Dirichlet boundary condition for the voltage and concentrations and behaves as a sink. Our simulations show that the close proximity of an ionic reservoir, such as the dendritic shaft or any large compartment, kills the signal, preventing it from propagating and penetrating nearby neuronal structures, such as thin branches at a bifurcation or dendritic spines. Conversely, a signal arriving at the leading edge of the dendritic tree, far from an ionic reservoir and where only small branches are present, will propagate at a greater distance and invade neighboring dendritic spines and branches.

This result provides insight into how structure is linked to function in dendritic spines. Indeed, although inducing synaptic plasticity at a synapse has been shown to change its structure, the question of spine compartmentalization is still being debated. While some studies suggest that the low resistance offered by the neck would not compartmentalize electrical activity (Svoboda et al., 1996), other experiments show the opposite Araya et al. (2006); Bloodgood et al. (2009). Our results suggest that the degree of compartmentalization of a spine can be determined by considering the distance between the spine and a large dendrite; a short distance leads to compartmentalization, while a long distance does not. We also propose that biochemical compartmentalization—the physical segregation of molecules such as calcium (Yuste et al., 2000)—can coexist with voltage invasion of a spine; that is to say, a spine can be a biochemical compartment without being an electrical compartment.

A way to test this prediction, would be to track electric and/or calcium activity in spines specifically. For instance, one could stimulate activity in individual spines via two-photon glutamate uncaging, record the activity of neighboring spines/branches and analyze the results in relation to the distance from the dendritic shaft or a major dendrite. Spines in the distal part of the dendritic tree should be less compartmentalized than proximal ones. This difference is more pronounced for voltage; therefore, tracking voltage dynamics in nearby spines should provide more accurate results. Note that calcium imaging could provide information on ionic compartmentalization, but not on electric compartmentalization. Note also that the time constant for concentration dynamics is in the order of seconds, whereas for voltage dynamics it is in the order of hundreds of milliseconds. This also impacts signal perception by nearby spines.

The main advantage of our method is that it provides a framework for accurately simulating highly non-linear systems in any geometry. This allows us to study the effect that finely tuned neuronal geometries have on their function. However, this comes with inherent difficulties, particularly with regard to the implementation and the development, which is why we use two-dimensional geometries. Simulations in three-dimensional geometries will be the subject of a future study, but we expect the qualitative results observed in this study to remain consistent. Note that the technical challenges of implementation also make the code difficult to share. A second difficulty concerns modeling, particularly investigating the plasticity rules at work in dendrites. In this study, we focus on a passive-membrane model and obtain qualitative results on ionic and voltage propagation in this context. To investigate plasticity more specifically, we will need to build a model that is representative of calcium dynamics in the dendritic tree. This requires including all ionic species in the PNP system (e.g., calcium, sodium, potassium, and chloride), which means adding a Nernst–Planck equation for each ion. The classical macroscopic approximation used in Cable theory, which eliminates the need to consider ionic concentrations, is no longer applicable. It also requires the addition of ionic channels, buffers, and pumps. Unfortunately, adding Hodgkin–Huxley channels is not straightforward, since the membrane cannot be considered as a simple capacitance in the PNP system. Finally, one possible development could be to derive an analytical approximation of our system. One way would be to use the framework of the sum-over-path approach in a one-dimensional graph model, including ionic channels as delta Dirac function for example, and to compare to our two-dimensional simulations.

Questions remain regarding the propagation of voltage and concentration in the dendritic tree, the linear or non-linear summation, and the role of the various channels, and our approach enables us to provide preliminary qualitative answers.

## Data Availability

The original contributions presented in the study are included in the article/supplementary material, further inquiries can be directed to the corresponding author.
